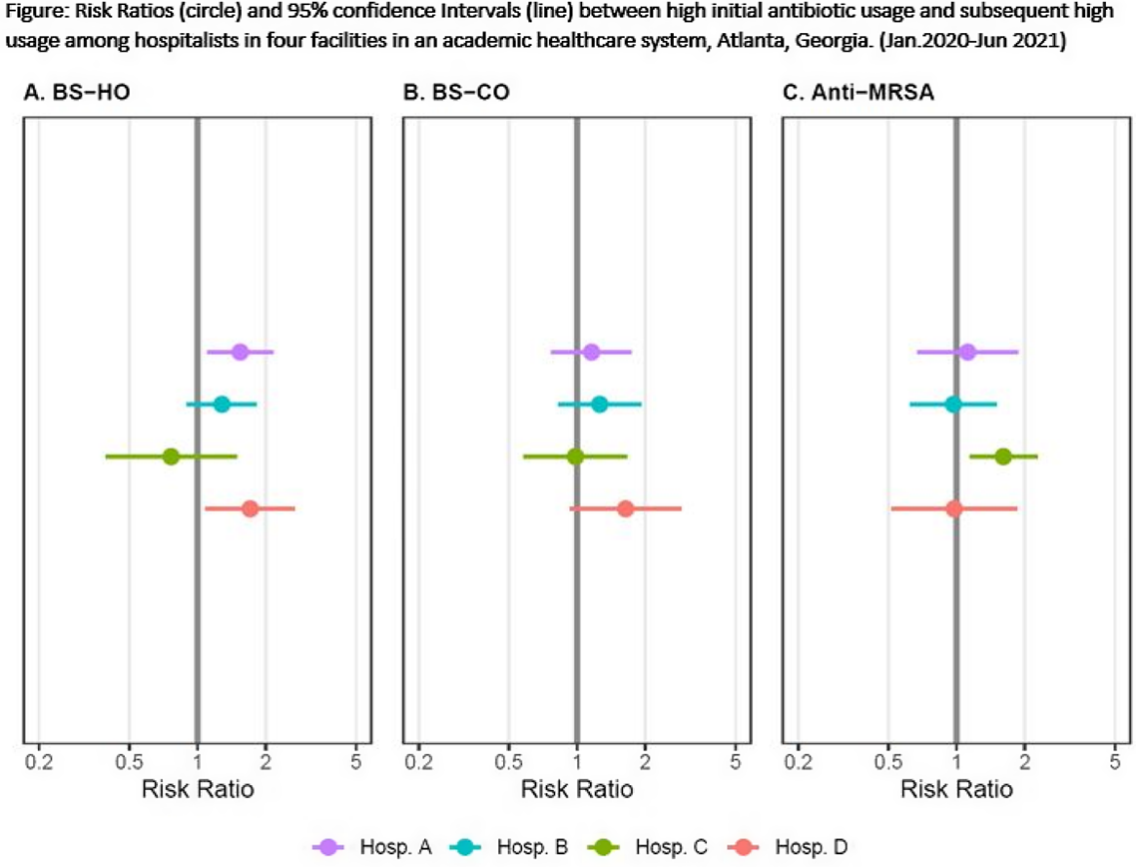# Do hospitalists who prescribe high (risk-adjusted) rates of antibiotics do so repeatedly?

**DOI:** 10.1017/ash.2022.58

**Published:** 2022-05-16

**Authors:** Udodirim Onwubiko, Christina Mehta, Zanthia Wiley, Jesse Jacob, Ashley Jones, Shabir Hassan, Marybeth Sexton, Sujit Suchindran, Scott Fridkin

## Abstract

**Background:** Provider-specific prescribing metrics can be used for benchmarking and feedback to reduce unnecessary antibiotic use; however, metrics must be credible. To improve credibility of a recently described risk-adjusted antibiotic prescribing metric for hospital medicine service (HMS) providers, we assessed whether providers who initially prescribed excess antibiotics continued to prescribe antibiotics excessively. **Methods:** We linked administration and billing data among patients at 4 acute-care hospitals (1,571 beds) to calculate days of therapy (DOT) ordered by individual hospitalists for each of 3 NHSN antibiotic groupings: broad-spectrum hospital onset (BS-HO), broad-spectrum community-onset (BS-CO), or anti-MRSA for each patient day billed from January 2020 to June 2021. To incorporate repeated measures by provider, mixed models adjusted for patient-mix characteristics (eg, % encounters with urinary tract infection, etc) were used to calculate serial, bimonthly, provider-specific, observed-to-expected ratios (OERs). An OER of 1.25 indicates that the prescribing rate observed was 25% higher than predicted, adjusting for patient mix. We then used log binomial generalized estimating equations to assess whether a high prescribing rate (defined as an OER ≥ 1.25) for an individual provider in an earlier bimonthly period was associated with a persistent high rate for that provider in the following period. **Results:** Overall, 975 bimonthly periods were evaluated from 136 hospitalists. Most (58%) contributed data the entire 18-month study period. Median OERs were similar between hospitals: 0.94 (IQR, 0.65–1.28) for BS-HO antibiotic use, 0.99 (IQR, 0.73–1.24) for BS-CO antibiotic use, and 0.95 (IQR, 0.65–1.28) for anti-MRSA antibiotic use. At the individual prescriber level, roughly one-quarter of bimonthly OERs (range varied by group and hospital from 21% to 31%) were categorized as high. At 3 of the 4 hospitals, a provider with a high OER for either BS-HO or BS-CO antibiotic use in any bimonthly period was more likely to have a high OER in the subsequent period (Fig. 1). These observed risk ratios were statistically significant for BS-HO antibiotic use at only 2 hospitals: hospital A risk ratio (RR) was 1.54 (95% CI, 1.10–2.16); hospital B RR was 1.28 (95% CI, 0.90–1.82); hospital C RR was 0.76 (95% CI, 0.39–1.48); and ospital D RR was 1.71 (95% CI, 1.09–2.68). **Conclusions:** Our findings suggest that hospitalists with a higher than expected 2-month period of antibiotic prescribing are likely to continue to have elevated prescribing rates in the following period, particularly for BS-HO antibiotics. These findings increase the credibility of using a 2-month prescribing metric for BS-HO antibiotic stewardship efforts; further work is needed to evaluate utility for other antibiotic groupings.

**Funding:** None

**Disclosures:** None

**Figure:**